# The Use of a Novel Virtual Reality Training Tool for Peritoneal Dialysis: Qualitative Assessment Among Health Care Professionals

**DOI:** 10.2196/46220

**Published:** 2024-08-06

**Authors:** Caterina Lonati, Marie Wellhausen, Stefan Pennig, Thomas Röhrßen, Fatih Kircelli, Svenja Arendt, Ulrich Tschulena

**Affiliations:** 1 Center for Preclinical Research, Fondazione IRCCS Ca' Granda Ospedale Maggiore Policlinico Milan Italy; 2 Fresenius Medical Care Bad Homburg Germany; 3 context Essen Germany

**Keywords:** peritoneal dialysis, virtual reality, patient education, patient training, chronic kidney disease, nursing, qualitative assessment

## Abstract

**Background:**

Effective peritoneal dialysis (PD) training is essential for performing dialysis at home and reducing the risk of peritonitis and other PD-related infections. Virtual reality (VR) is an innovative learning tool that is able to combine theoretical information, interactivity, and behavioral instructions while offering a playful learning environment. To improve patient training for PD, Fresenius Medical Care launched the *stay•safe* MyTraining VR, a novel educational program based on the use of a VR headset and a handheld controller.

**Objective:**

This qualitative assessment aims to investigate opinions toward the new tool among the health care professionals (HCPs) who were responsible for implementing the VR application.

**Methods:**

We recruited nursing staff and nephrologists who have gained practical experience with the *stay•safe* MyTraining VR within pilot dialysis centers. Predetermined open-ended questions were administered during individual and group video interviews.

**Results:**

We interviewed 7 HCPs who have 2 to 20 years of experience in PD training. The number of patients trained with the *stay•safe* MyTraining VR ranged from 2 to 5 for each professional. The *stay•safe* MyTraining VR was well accepted and perceived as a valuable supplementary tool for PD training. From the respondents’ perspective, the technology improved patients’ learning experience by facilitating the internalization of both medical information and procedural skills. HCPs highlighted that the opportunity offered by VR to reiterate training activities in a positive and safe learning environment, according to each patient’s needs, can facilitate error correction and implement a standardized training curriculum. However, VR had limited use in the final phase of the patient PD training program, where learners need to get familiar with the handling of the materials. Moreover, the traditional PD training was still considered essential to manage the emotional and motivational aspects and address any patient-specific application-oriented questions. In addition to its use within PD training, VR was perceived as a useful tool to support the decision-making process of patients and train other HCPs. Moreover, VR introduction was associated with increased efficiency and productivity of HCPs because it enabled them to perform other activities while the patient was practicing with the device. As for patients’ acceptance of the new tool, interviewees reported positive feedback, including that of older adults. Limited use with patients experiencing dementia or severe visual impairment or lacking sensomotoric competence was mentioned.

**Conclusions:**

The *stay•safe* MyTraining VR is suggested to improve training efficiency and efficacy and thus could have a positive impact in the PD training scenario. Our study offers a process proposal that can serve as a guide to the implementation of a VR-based PD training program within other dialysis centers. Dedicated research is needed to assess the operational benefits and the consequences on patient management.

## Introduction

### Background

Compared with in-center dialysis, peritoneal dialysis (PD) confers significant benefits to patients with chronic kidney disease (CKD), including better preservation of residual renal function and higher treatment flexibility [1,2]. As a result, kidney health organizations recommend facilitating and increasing patients’ access to home dialysis [3]. The SARS-CoV-2 pandemic emphasized the importance of expanding PD use [4]. However, only 11% of patients on dialysis are currently treated with PD [5]. Poor patient education and inadequate training were identified as significant factors contributing to the global underuse of PD [6,7]. In line with this, patients who received structured training more likely chose home dialysis over in-center treatment [8]. In addition, in a telemedicine patient education study, patients with CKD stages 4 and 5 receiving telemedicine predialysis education had increased health literacy and increased home modality choice [9].

Besides learning theoretical concepts, patients with CKD need to acquire physical skills to perform the PD procedure by themselves. Therefore, PD training programs mainly involve individual sessions between patients and nephrology health care professionals (HCPs) [10,11], with most of them represented by nurses [11]. According to an international survey, a successful PD training program typically requires an average training time of 30 hours or 6 days per patient and is predominantly conducted in a one-to-one setting with both the patient and the nurse [11,12]. Increasing evidence demonstrated that efficient patient training can also lead to improved patient outcomes [13-15]. In fact, longer training time was associated with lower PD-related peritonitis rates [13,15], while frequent patient retraining reduced the risk of exit-site infections [14]. Consistently, the International Society for Peritoneal Dialysis guidelines recommended PD training standardization and optimization to reduce peritonitis rates [16]. On the basis of these observations, the International Home Dialysis Roundtable prompted the adoption of new strategies to improve education programs and boost patient engagement in training activities [3]. Of interest, the use of visual or audio aids and computer-assisted instructions was proposed to enhance patient learning [17].

To meet these requirements, Fresenius Medical Care (Bad Homburg, Germany) launched a novel training program for continuous ambulatory PD (CAPD) based on the use of virtual reality (VR), referred to as *stay•safe* MyTraining VR [18]. VR is an innovative technology with a huge potential in medical training [19-25] and patient education [26-32]. VR uses head-mounted displays to create an immersive, computer-generated, 3D, and interactive environment. *Stay•safe* MyTraining VR equipment includes a VR headset and a handheld controller designed to support the training of patients on hygiene procedures, preparation and posttreatment steps, and bag exchange and operation. The novel digital training tool was introduced to support the classical PD training at 3 dialysis centers in Germany. As for the traditional PD training, nurses and nephrologists continue to be responsible for patient education when being supported by the *stay•safe* MyTraining VR.

### This Study

This qualitative research investigated, for the first time, HCPs’ perspectives, preferences, and attitudes toward the implementation of the *stay•safe* MyTraining VR within CAPD training. We sought to describe how and when the tool was integrated in the traditional training framework and to evaluate whether VR introduction had an impact on patient learning. Moreover, HCPs’ opinions about the potential benefits and limitations related to VR were explored. Professionals’ and patients’ acceptance of the new technology was likewise investigated.

## Methods

### Study Design and Study Sample

This study is an integrative qualitative research involving convergent, narrative, problem-centered, and discursive interviewing designed to investigate the following aspects: (1) integration of the new tool into the classical PD training framework and professionals’ perspectives on its usability, (2) target patient groups who can benefit from the new technology, (3) benefits and advantages, (4) limitations and weaknesses, (5) HCPs’ and patients’ acceptance of the new technology, and (6) additional applications. The collected information was then used to derive a process through which VR training can be implemented in the conventional training curriculum. All nursing staff and nephrologists in the 3 NephroCare dialysis centers where this technology was piloted, who already gained practical experience with the *stay•safe* MyTraining VR, were invited by mail to the respective medical administration for voluntary participation. Overall, 6 nurses and 1 nephrologist agreed and participated in this study.

### Ethical Considerations

All participants read and signed the informed consent document, including a privacy policy explaining data collection, data use, and data storage, before participating in the study. To ensure confidentiality, all participant data were anonymized before analysis. No compensation was provided to the participants for their involvement in this study. Participants were free to withdraw from the study at any time and were informed so, and participation was carried out on a voluntary basis.

This study did not undergo formal ethics review. This is justified based on the anonymity of responses and because no risk was expected to survey participants and basic ethical principles (individual autonomy, self-determination, avoidance of harm, care, and justice) were not violated, as mentioned by the ethics commission of Bavarian universities (Gemeinsamen Ethikkommission der Hochschulen Bayerns [33]), where it is outlined that no ethics commission review is needed when no risk of damage is to be expected for participants and if basic ethical principles are not violated. As an example for such an exemption, a questioning of experts is mentioned, as this does not cause any particular risk or burden beyond what is witnessed in everyday life, as in this study.

### Data Collection

To maximize information gathering, the interviews included open-ended questions and were conducted digitally as individual and group video interviews.

More specifically, the interviews included the following phases (Multimedia Appendix 1): (1) introduction and transparency of objectives, approach, and methodology; (2) introductory question for topic identification and prioritization by the interviewee; (3) narrative phase with nondirective (only encouraging) interventions by the interviewer; (4) discursive phase, in which the interviewee is questioned about their views, opinions, and evaluations and is asked to analyze and reflect in greater depth; and (5) open guiding questions according to the interview guide.

During the discursive phase, initial hypotheses were confirmed, concretized, adjusted, or rejected in an iterative process through repeated testing and feedback. To this aim, different forms of intervention were used: request for justification, explication of gaps and contradictions, validation of conclusions, target-actual comparison, and hypothesis-guided and solution-oriented questions.

All the interviews were conducted by SP and TR, took approximately 60 to 90 minutes each, and were recorded in writing.

### Definitions

VR “effectiveness” was investigated considering the following aspects: (1) patient satisfaction (question 1: what is the feedback from patients? question 2: what is the range here?), (2) patient learning success (question 1: how do you rate the learning success in comparison to classic training? question 2: where is it greater, where less?), and (3) risks related to the use of the VR technology in the PD educational setting (question 1: what risks do you see in a full transition to VR?)

By contrast, “efficiency” involved the evaluation of (1) time committed by HCPs to present the technology to patients (question 1: how does your time commitment compare to traditional training?), (2) ratio of effort to benefit (question 1: is the cost-benefit ratio appropriate?), and (3) patient throughput (question 1: can you care for more patients with the new method?)

### Qualitative and Statistical Analyses

A category system (Multimedia Appendix 1) for the core statements was inductively formed based on the material so that frequency distributions of topics or core statements and the associated evaluation were possible (descriptive statistics). The results were compressed, structured, and interpreted by means of a qualitative content analysis.

We computed the absolute value and relative frequency for categorical variables.

## Results

### HCPs’ Characteristics

A total of 7 HCPs were interviewed, of whom 5 (71%) were nurses and 2 (29%) were nephrologists. All the respondents had >10 years of work experience with patients with CKD. HCPs had 2 to 20 years of experience in PD training, with an average of 1.5 months of experience with the *stay•safe* MyTraining VR. The number of patients trained using the VR technology ranged from 2 to 5 for each professional.

### HCPs’ Use of the VR Technology Within the PD Training Program

**Figure 1:**
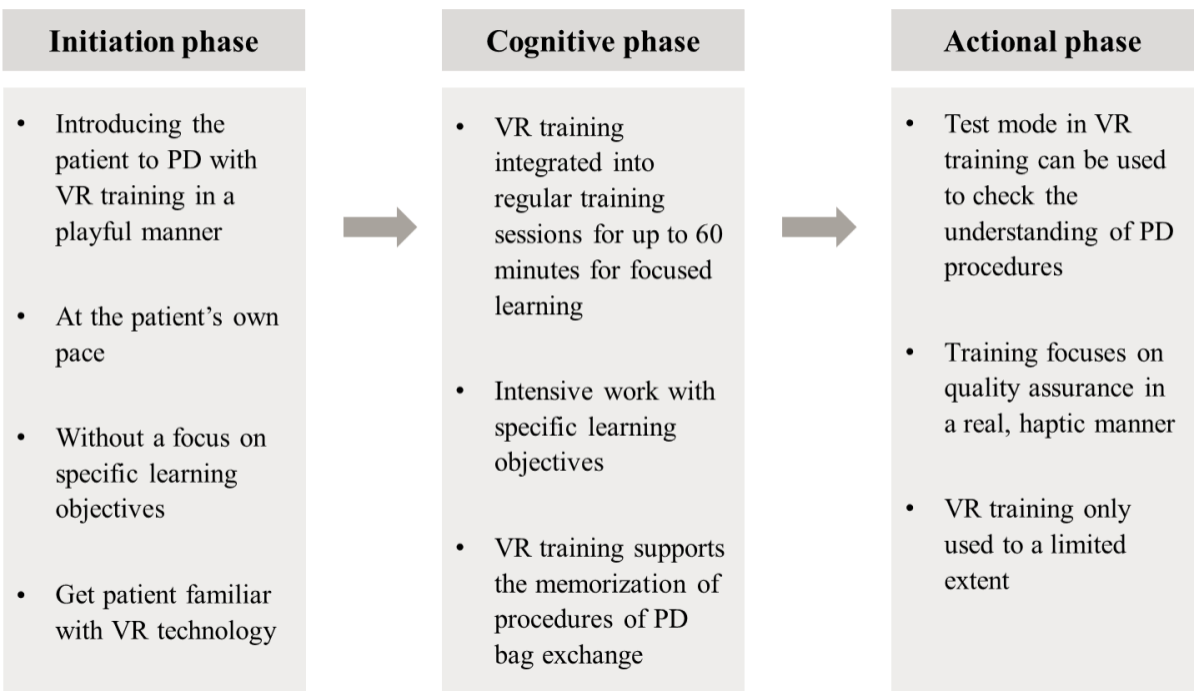
Use of the Stay•safe MyTraining virtual reality (VR) within the classical workflow of patient education for home dialysis. According to the interviewed health care professionals (HCPs), peritoneal dialysis (PD) training involves three consecutive phases: (1) motivational phase, where patients are instructed with the basic information about PD without excessive pressure due to initial emotional load; (2) cognitive phase, where patients acquire all the theoretical concepts as well as the procedures to perform home dialysis alone; and (3) actional phase, where patients practice using the various materials and consumables and HCPs check whether the different learning objectives are effectively achieved. HCPs reported a successful integration of the novel VR tool into the routine activities during the motivational and cognitive phases. Notably, the VR training provided substantial advantages and benefits, allowing the improvement of patients’ learning experience. By contrast, the use of the VR tool was limited in the actional phase because the acquisition of the practical skills required a real, haptic experience with the various materials and aids.

HCPs believed that the VR technology was a valuable complementary tool to the traditional PD training program. On the basis of the collected statements, the classical PD training takes approximately 2 weeks and comprises 3 different phases (Figure 1). It typically begins by providing patients with essential information needed to get a basic understanding about how PD works and how it will impact their lifestyle and habits. This process imposes a significant emotional overload on patients (motivational phase). Next, patients are instructed with all the theoretical concepts needed to successfully perform home dialysis (cognitive phase). Finally, once basic knowledge is acquired, patients start practicing the procedures using the dedicated materials and consumables. In this phase, HCPs assess patients’ understanding, detect eventual risks or mistakes in performing the different tasks, and provide instructions to successfully manage any critical events (actional phase).

According to the interviewees, the novel approach was easily integrated into these routine training activities. Timings and modalities of VR administration were selected by each professional based on individual patient characteristics, receptivity, and interests. More specifically, our analysis indicated that the respondents used the VR tool with different aims depending on the particular training phase. For instance, after catheter insertion, VR was frequently offered to help patients approach PD in a playful manner, without immediately focusing on specific learning objectives:

I think the tool is especially good for providing systematic information and orientation to strengthen compliance to treatment.

The VR tool was particularly helpful during the cognitive phase. Indeed, the use of VR enabled HCPs to more effectively explain the relevant instructions and to make the procedures clearer, facilitating patients’ internalization of both cognitive and procedural skills. Hence, most of the interviewees thought that the VR technology significantly improved patient learning experience by enabling patients to anchor learning success, process routine, procedural safety, and efficiency in home dialysis. HCPs believed that at this training stage, the VR-based approach could replace parts of the classical program:

VR is a focused learning medium that can be used when the patient has established orientation and acceptance [for the therapy]

It’s probably not only good for decision making, but maybe also for learning the basic home dialysis process in more depth.

VR can serve as a support, it facilitates learning...But with VR, we have a different learning level. As a patient, you have to work it out for yourself. The training serves to deepen the knowledge and sets a different focus...With the VR glasses you can internalize the sequence, that is what it is good for, that is what this technology supports.

By contrast, HCPs reported limited use of the VR technology during the actional phase, when patients need to perform tactile dialysis-related tasks using all the equipment or consumables in sterile conditions. In fact, in the view of the interviewees, the VR technology could not replace classical training during this final phase because safe handling requires real experience with the various materials and aids. In addition, HCPs believe that error prevention and management as well as “quality control” need to be achieved through classical one-to-one sessions:

VR training does not replace classical training. In any case, the patient has to learn well the handling during bag change even without VR training. VR training is a supplement.

The patient should first see the practical procedures such as turning on the exercise material first. Only then can VR be used. However, the handling still has to be experienced haptically.

After the virtual trial, he then has to perform it independently in real life under observation.

Definitive coverage with VR training is not possible. However, it can be a large component. But you also need a haptic test in real life.

### Target Patient Groups for VR Training According to HCPs

In the interviewees’ opinions, the VR technology was suitable for most patients, including older adults. Patients’ basic motivation and willingness to engage in the VR technology were indicated as essential prerequisites for the success of VR-based training:

There are no clear patient groups that you can include or exclude...Mental flexibility is crucial.

The use of VR technology is not age-dependent. The interest depends on the personal attitude.

PD may also be used in patients with limited capabilities in some cases. This was also addressed by the respondents in the study, but overall, the use of VR training was not considered suitable for patients with dementia. Limited use with patients experiencing severe visual impairment or lacking sensomotoric competence was also mentioned:

VR training is basically possible for almost all patients, but the key is how the patient receives it. Only in patients with dementia VR training is not applicable.

The glasses are not suitable for participants with severe visual impairment because they cannot see and recognize sufficiently. A younger colleague with impaired vision was also unbale to see sufficiently in the glasses here.

### Benefits and Advantages of VR Training

VR training was perceived as a tool able to improve cognitive learning effects and make the training process more efficient in terms of personnel and material resources. The advantages and strengths of the technology pointed out by HCPs are listed in Table 1.

First of all, according to responders, VR introduction into PD training facilitated patient education and improved learning experience. First, VR training allowed patients’ immersion into a focused learning environment and provided an efficient shielding from external stimuli (point 1, Table 1). This maximized patients’ engagement in the different educational activities. Second, VR offered learners the opportunity to repeat training activities according to their individual needs (point 2, Table 1). This enabled patients to correct errors and learn a standardized curriculum in a safe learning environment. Training session repetition allowed learners to get familiar with the use of dialysis equipment and supplies, boosting their confidence in performing the specific tasks. Third, HCPs noted that by providing multiple learning stimuli, VR-based training improved the efficiency of knowledge transmission (point 3, Table 1). In addition, the creation of a playful learning environment contributed to enhancing patients’ engagement and, consequently, to improving their learning experience while avoiding overwhelming them with too much information (point 4, Table 1).

Besides facilitating patients’ learning process, the use of technology conferred different benefits to HCPs (point 2, Table 1). In fact, VR helped professionals explain the operating principles of PD, allowing them to convey the information in a simplified and intuitive manner (point 5, Table 1). Moreover, because HCPs were able to perform different activities (point 7, Table 1) while the patient was practicing with the devices, VR was perceived as a significant tool to increase their productivity (point 6, Table 1). Nurses also reported that VR-based training enabled an individual operator to care for more patients at the same time due to higher training efficiency (point 7, Table 1). In the HCPs’ view, another relevant advantage provided by the VR-based training was the opportunity to standardize the educational program, following the systematic content of the VR software (point 10, Table 1).

Finally, as the virtual experience does not require the use of additional PD supplies, the costs of training can be significantly reduced, as the number of dialysis fluid bags needed for training can be reduced (point 11, Table 1).

**Table 1:** Benefits and advantages of virtual reality (VR)–based training according to the interviewed health care professionals (HCPs).

Benefits and advantages		Representative quotes
**For patients**		
	1. Focused learning environment	“In VR, the patient has no distractions, but is completely concentrated in the system. Those who like to move around in this system and are focused in it learn quickly.” “In conventional training, patients are very distracted by stimuli from their immediate environment and their minds are then elsewhere. In VR training, for example, you do not see the nurse who suddenly comes into the room and disturbs you.” “VR can serve as a support, it facilitates learning...But with VR, we have a different learning level. As a patient, you have to work it out for yourself. The training serves to deepen the knowledge and sets a different focus...With the VR glasses you can internalize the sequence, that's what it’s good for, that’s what this technology supports.”
	2. Unlimited repetition of the procedures	“The patient then repeats it as often as he wants and until it works.” “It is good for the mindless rehearsal of the procedures. The patient then repeats it as often as he wants and until it works. This is a learning effect.” “More security is created because the process is replayed over and over again.”
	3. Multiple learning stimuli	“With VR, we have a different learning level. As a patient, you have to work it out for yourself. The training serves to deepen the knowledge and sets a different focus.” “The VR training can connect well to the therapy concept in the center to also bring variety into the training.”
	4. Playful learning environment	“VR mainly serves to support the theory in a playful way, not doctrinal, but a different approach, not like an exam.” “The VR is playful, not schoolmasterly. It’s a different approach with less pressure.” “VR takes the pressure out of the learning process.”
**For HCPs**		
	5. Simplified knowledge transfer	“In classic training, I do a lot of words and that may then lead to mental shutdown. VR shows that in a much shorter time.” “The training replaces what I do verbally otherwise. Important aspects like turning off air conditioning, pets out of the room, etc. they see in the movie.”
	6. Enhanced HCPs’ efficiency or productivity	“The training replaces what I do verbally otherwise. Important aspects like turning off air conditioning, pets out of the room, etc. they see in the movie. In classic training, I do a lot of words and that may then lead to shutdown. VR shows that in a much shorter time.” “The patient had good observation skills...Therefore, I was only fully present the first time. The second time, I just put on my glasses and was gone for 15-30 minutes. I went to another patient and she stayed alone in the training room.”
	7. More effective time management	“We can perform the following activities in parallel, for example, in the same room in the presence of the patient: Prepare laboratory for the next patient, evaluate laboratory tests, calculate peritonial equilibrium test (PÄT), write prescription, arrange appointments, sort and file findings, etc...” “I can imagine that you can still do something in parallel in the same room at the desk (e.g., look at lab values, do documentation, prepare classic PD, e.g., tear open bags).” “The patient had good observation skills...Therefore, I was only fully present the first time. The second time, I just put on my glasses and was gone for 15-30 minutes. I went to another patient and she stayed alone in the training room.” “I can do other things in parallel, there I have a discretion. There would be further efficiency if several patients are cared for in parallel in one room with VR goggles. I would try this.” “Many parallel/routine activities in the room are possible, which are not possible in classical training.”
	8. Induction and motivation of HCPs	“New young non-specialized nursing staff, can get orientation via the VR. One can also see in the VR glasses a motivational approach for employees who do not yet have a connection to dialysis or home dialysis and are in training or further education. They can then realize ‘oh, this is a very interesting area of work for me.’” “The VR training is useful for the introduction of staff, including trainees and physicians.”
	9. HCPs’ training	“It is good for nurse training, doctor training and patient training.”
**For dialysis centers**		
	10. Standardization of training programs	“The VR technique is not so subjective in its application. The program allows objectification, unification and a standardized approach. One forgets then nothing.”
	11. Cost saving	“Material costs: The number of bag exchanges is reduced. One consumes 11-15 € per training. You can save about 5 bags.”

### Limitations and Weaknesses of VR Training

A list of the potential drawbacks of VR training is provided in Textbox 1.

According to HCPs, the most significant weakness of VR training resides in the inherent inability to provide learning effects in the area of tactile perception and sensorimotor fine control (point 1, Textbox 1).

Moreover, the traditional approach was still perceived as the best strategy to manage the emotional aspects related to PD treatment and address any patient-specific educational needs. In fact, according to the interviewees, the establishment of a trusting relationship between HCPs and learners is an essential prerequisite to identify emotional barriers and motivational obstacles toward PD (point 2, Textbox 1). Despite being indicated as a tool to reduce HCPs’ workload, some respondents were concerned about a possible additional workload to acquire the concept of VR training both in their curriculum and for their patients during the first VR-based training sessions (point 3, Textbox 1).

**Drawbacks and weaknesses of virtual reality (VR)–based training according to the interviewed health care professionals (HCPs).**

**Drawbacks, weaknesses, and representative quotes**

1. Lack of tactile education
  “It’s difficult in VR technology to get that shown virtually with the dressings. You don’t get a feeling for the catheter connection in VR, for example the resistance when screwing it shut.”
  “VR cannot replace the haptic real-world experience.”
  “VR training does not replace classical training. In any case, the patient must learn well how to handle the bag change even without VR training. VR training is a supplement and VR training does not replace classical training. In any case, the patient must learn well how to handle the bag change even without VR training. VR training is a supplement.”
  “...the handling still has to be experienced haptically...VR is not a substitute for traditional training.”
2. Relationship with the patient remains important
  “The nurse cannot be replaced by the device. The events and intermediate questions must be clarified. There are quite spontaneous questions.”
  “The relationship aspect of care is indescribably important. VR does not change that.”
  “The device is a supplement, the nurse cannot be replaced by the device. The events and intermediate questions must be clarified. Questions arise spontaneously.”
  “It’s all schematic in VR training. Unusual events can’t be handled in the system.”
  “Everything is schematic. Unusual events cannot be processed in the system. There are typical critical events that are not mapped.”
3. Initial workload for HCPs
  “The time for preparation and implementation of VR technology is then missing in the dialogue with the patient.”
  “In the beginning you have some effort, but only later there is a time effect.”

### HCPs’ Perspectives, Motivation, and Attitudes Toward the VR Technology

An investigation of respondents’ attitudes toward VR revealed some differences among HCPs (Textbox 2). A large proportion of professionals was willing to use the new tool but felt the need to first gain self-confidence in using the technology. Some HCPs found VR exciting and were interested and enthusiastic. By contrast, others showed a nonpositive feeling and distrustful attitude toward the tool.

**Health care professionals’ (HCPs’) perspectives, motivation, and attitudes toward the virtual reality (VR) technology.**

**HCPs’ attitudes and representative quotes**

Positive

- “The VR training can connect well to the therapy concept in the center to also bring variety into the training.”
- “I find the technology innovative.”
- “You also have to see it as a technology of the future. I think it is good when things develop. I’m fundamentally interested and open.”

Open to learn

- “I have to develop confidence first.”
- “There is probably already an acceptance in the team, but for me it means a learning curve. The trust in VR has to develop first.”
- “The first attempt was very confusing for me. Each subsequent one went better each time.”
- “I find the VR application basically exhausting, but interesting. I find it fascinating.”
- “VR is still a bit awkward to use: to grip the disinfectant, you have to use your hand, but before that you have to do something else...Other than that, I’m thrilled.”
- “In the beginning you have some effort, but only later there is a time effect.”

Negative

- “...there are also employees who only have a smartphone but no other IT skills. They tend to reject it.”

### VR Acceptance by Patients According to HCPs

In HCPs’ opinions, patients’ feedback on VR training was overall positive, and the acceptance was high, including that of older patients:

One patient [aged 82 years] has always wanted to exercise, but has not felt that well yet. VR is appropriate for him because he is really interested, because he really wants to learn. He thought it was great, is open minded.

...we initially dealt with the topic of home dialysis rather “playfully.”...She [older patient aged approximately 75 years] found the VR training enjoyable and interesting.

Of note, HCPs were aware of the importance of adopting a patient-tailored approach to present the technology. In fact, the respondents highlighted the need to determine the patient’s learning style and then to provide an initial clear and simple explanation of the use and purposes of the VR technology:

VR training is basically possible for almost all patients, but the key is how the patient receives it.

You first have to check whether it makes sense, taking into account native language and level of education, acceptance of the medium, etc.

You have to think carefully about when exactly to use the VR. The patient first has to get to know a lot of the basics and deal with the PD. Then, the patient needs a technical briefing: How does VR work? How do the individual elements such as the controller, etc. work? Then you first have to do 2-3 learning units and then a certain habit develops.

HCPs reported a lower VR acceptance among patients not familiar with electronic or audiovisual media and devices, irrespective of their age:

Not many, but some find it awful if there was no computer experience. One patient did not understand it properly.

People who are inexperienced with computers find it difficult to use them.

### Additional Applications of VR Training

HCPs believed that, besides patient education, the VR technology can be applied with other relevant goals and functions in the context of PD.

The administration of VR training before obtaining informed consent could significantly support patients in their decision-making about home dialysis. In fact, allowing an in-depth understanding of the PD procedures, VR training can improve patients’ awareness and understanding about home-based treatment and, consequently, help them take more informed decisions:

The VR glasses can be used at the beginning in the patient consultation before the treatment decision is made...It can support the patient’s decision making for home dialysis.

At a later stage, VR can be used as a motivational tool to enhance and reinforce patients’ decision about home PD:

If it is clear that the patient needs dialysis, then you can explain different procedures to them and then for explaining PD you can use VR. That is great.

In addition, HCPs believed that VR could be offered to patients’ family caregivers, who often play an important role in patients’ decision-making as well as in their care activities:

VR training can also be used for training of family members.

Another field of application of VR training could be within educational programs addressed to the HCPs themselves. Target users include both professionals already working with patients with CKD, such as nurses from cooperating ambulatory care services, and HCPs who are not directly involved in the PD process:

The VR training is useful for the introduction of staff, including trainees and physicians. VR training can also be used as training for family members. Retirement homes: nursing staff there, are yes very tightly staffed. The VR training can also be used in nursing homes and in hospitals with nursing staff for further training purposes.

*It is good for nurse training, doctor training and patient training.*

## Discussion

### Principal Findings

This qualitative assessment shows that according to nephrology professionals, the *stay•safe* MyTraining VR may significantly improve PD training efficiency and efficacy. In fact, VR was perceived as a valuable complementary tool able to enhance patients’ learning experience by providing the experiential learning necessary for a deeper understanding of medical information. In addition to the positive effects for patients, the *stay•safe* MyTraining VR may also provide HCPs with the opportunity to improve their productivity, both by saving their time and by facilitating the transmission of medical information to patients.

VR is an immersive experiential technology that emulates the physical world through digital simulation. Although originally intended for entertainment purposes, VR has a huge potential as a learning tool due to its unique ability to combine procedural information, interactivity, acoustic and visual information, and behavioral instructions [34]. In the medical field, VR-based education opened a new era of professionals’ training on surgical and endoscopy techniques [19-25,35]. More recently, the VR technology has also been implemented for patients’ education and training [26-32]. An increasing number of studies demonstrated that the use of simulation media can support different patients’ learning styles by providing a mix of visual, auditory, interactive, and text elements [29,30] and improve patients’ understanding and comprehension of their diseases as well as of the treatment or lifestyle interventions required to cope with them [26,29,32,36-38]. In addition, by enabling the repetition of training sessions according to individual patient needs, the technology facilitates the acquisition of competences and skills by learners. The possibility to build muscle memory through experiential training [30] and to learn from mistakes in a safe environment further improves patients’ learning experience. VR is similarly emerging as an innovative strategy to foster self-management and self-care in patients with chronic conditions [28]. Finally, VR-based education could increase patient engagement and empowerment [26] while reducing anxiety and pressure related to medical procedures [26,39-41].

In nephrology, effective patient education and training have an impact on the risk of peritonitis [13-15]. In fact, training for home therapies poses unique educational challenges, as patients need to acquire not only the theoretical concepts of dialysis but also the technical skills required to manage all the procedures on their own [2,42]. Recently, Zgoura et al [43] proposed the use of VR headsets and gamification elements in support of PD training with the aim to standardize, facilitate, and accelerate patients’ learning process. Here, we report HCPs’ perspectives, opinions, and attitudes toward the implementation, into PD training programs, of the *stay•safe* MyTraining VR, a novel PD training program based on the use of a VR headset and a handheld controller [18]. We first explored the timings and modalities of VR integration within the classical training curriculum. Overall, the VR technology was perceived as a helpful supplementary tool, but HCPs specified some functional differences between the traditional and VR-based training programs. According to HCPs, VR-based training was particularly useful during the cognitive phase of the PD training program, where it not only enhanced patients’ learning experience and facilitated information internalization but also assisted nurses in explaining the theory and procedures in a more effective and simplified manner. VR was a valuable support also during the initiation phase, during which it helped HCPs overcome potential patients’ emotional barriers and lack of self-confidence. Conversely, according to the respondents, the actional phase still required a more classical approach, as a real, haptic experience was indicated as essential to learn the correct handling of the various materials as well as how to manage any critical steps or mistakes.

In HCPs’ opinions, a significant added value of the VR technology resides in its ability to improve patients’ learning experience and make the entire training process more effective and efficient. These relevant effects are achieved, thanks to the inherent characteristics of immersive or interactive media, which were clearly identified by the interviewees, including a focused learning environment and multiple learning stimuli. The opportunity to repeat the training modules based on patients’ individual needs and cognitive skills was also highlighted. The impact of task repetition in education and training is currently well known [17,44]. In the context of health care educational interventions, the repetition of training activities resulted in faster skill acquisition and improved transfer of learning to practice [45]. The mechanisms underlying repetition benefits in cognitive learning include the induction of long-term memory [46] and internalization of procedures as unconscious habits [47]. With each repetition, the cognitive effort required for memory performance, behavior planning, and action control is reduced [30]. This allows rapid recall of habits from memory and improves learners’ performance and confidence. Moreover, HCPs noted that repeated training sessions can offer patients the opportunity to correct errors and refine their skills in a safe learning environment. Therefore, patients could get instructions and learn from their mistakes. Learning by doing is one of the central features of interactive education because it allows acquiring a high level of experience and becoming accustomed to the therapy or procedure [17,25,48]. The playful and interactive learning environment may increase motivation in PD training. As shown by Kyaw et al [20], VR interventions with more interactivity showed better results in terms of knowledge and skills outcomes than interventions with less interactivity. Therefore, the *stay•safe* My Training VR, as a highly immersive training program, may lead to higher patients’ motivation and, consequently, increased learning success. These unique attributes of VR could bring substantial benefits especially for older adults, who often need more training time to learn the procedure of PD [42].

Besides the positive effects on patients’ cognitive learning, HCPs identified distinctive benefits associated with the use of *stay•safe* MyTraining VR as an informative tool. In fact, VR helped HCPs to illustrate the home dialysis process to patients without providing them with too much stressful information. Similar results were obtained with the use of immersive media to prepare patients for medical procedures [32] and radiotherapy [37] and to increase patients’ knowledge about their disease and its associated consequences [26,29].

Concerning patients’ acceptance of the new tool, HCPs reported that most of them showed a positive attitude toward VR. These observations confirm and expand the results provided by previous studies in populations with CKD, in which the use of VR either as a distraction or an educational tool was associated with high levels of satisfaction [31,49-51]. On the basis of the HCPs’ statements, there was no difference in patients’ engagement in the VR-based training between older and younger people. Consistently, recent research in participants with abdominal aortic aneurism and stroke indicated that patients’ age does not affect their engagement in VR-based educational programs and is not associated with cybersickness [32,52,53]. As CKD is becoming more prevalent in older individuals [54], these observations appear particularly relevant for a future implementation of the VR training tool into PD education. By contrast, HCPs noted that people who were not familiar with the use of electronic or audiovisual devices tended to build emotional barriers or showed a greater uncertainty in their use of VR. These findings are in line with the studies performed by Specht et al [52] and Huygelier et al [53], who found that patients with a negative attitude toward electronic media were more likely unwilling to use VR. Psychological research showed that acceptance toward computer media is significantly influenced by users’ perceptions of the ease of use, usefulness, and playfulness of the different tools [55,56]. Of interest, studies conducted among older adults showed that users’ perception that VR was useful, easy to use, and fun promoted a positive attitude toward the tool [57,58]. Moreover, interactivity was indicated as an important factor influencing the intention of continuous use of virtual practices [59]. Therefore, recommendations to boost patients’ initial motivation toward VR include maximizing the positive aspects through increasing interactivity, enhancing users’ perceptions of utility, reducing the difficulty associated with its use, and enhancing the playful nature of the training.

In addition to the several advantages for patients with CKD, the interviewees were able to identify different positive effects on their working activity. Indeed, in the HCPs’ experience, professional assistance during VR-based training became increasingly unnecessary once patients acquired the skills to use the technology. Thus, HCPs were able to focus on numerous parallel activities and routine administrative tasks during training sessions. This allowed more effective time management among nurses [60]. Moreover, the respondents reported that by providing simple and schematic contents, VR helped convey the medical information more easily. This facilitated HCPs’ teaching activity and contributed to boosting patient engagement in PD training. Consistent with these observations, professionals’ acceptance toward the new tool was overall high. However, many employees highlighted that they felt the need to undergo structured training to get familiar with VR before using the technology with patients. Therefore, confidence in using the VR technology must be built up to recognize and use its full potential.

Another important improvement offered by VR-based PD training is related to cost-effectiveness. In fact, as the virtual experience does not require the use of additional dialysis fluid bags for mock training, the costs required to successfully train each patient in performing PD can be reduced [43]. Given the increasing prevalence of CKD [61], a more effective health care resource use is essential to ameliorate patient care. By providing the opportunity to save money on dialysis supplies and improving nurses’ time management, VR-based training could contribute to reducing the economic burden of PD training while ensuring a high-quality educational offer.

HCPs mentioned other possible applications of VR beyond direct patient preparation for home dialysis that have not yet been tested in practice. The *stay•safe* MyTraining VR was used within the educative conversation with people who need to start dialysis with the aim to enhance the visibility of home therapies as a treatment option and to prepare patients psychologically and emotionally for PD. In this context, VR can support patients to make informed decisions about their treatment of choice. Of note, the gaming factor could have a crucial role in reassuring patients and boosting their self-confidence. Barriers for home dialysis training could thus be overcome by using VR as a complementary tool, which may in turn lead to increased uptake of home dialysis. HCPs also proposed to take advantage of the VR tool to inform patients’ relatives about CAPD as a treatment option. As patients’ and caregivers’ low awareness about and poor understanding of home therapies are important factors underlying the low uptake of PD [6,62], the use of the VR technology as an informative motivational tool can have an important clinical impact among patients with CKD.

Some drawbacks related to the technology were also highlighted by HCPs. In the respondents’ view, VR’s inability to provide real, tactile education limits its use during the final phase of the PD training program. At this stage, while patients need to practice the procedural skills required to complete each task, HCPs check patients’ ability to perform the procedures independently and manage eventual critical steps or mistakes. Therefore, the conventional approach was perceived as the best option for both patients’ skill acquisition and professionals’ quality assurance. The respondents similarly identified a potential limited use of VR training with specific patient groups, mainly among people experiencing cognitive disabilities or visual impairment.

Overall, the collected information indicates that VR implementation into clinical practice may have a profound impact in the PD training scenario. In fact, increasing patients’ proficiency in self-management may lead to improved outcomes among patients with CKD [63] and to lower patient concerns related to home dialysis procedures, thereby improving their quality of life. This has a great relevance considering the high burden of anxiety in patients receiving PD [41]. Moreover, the use of VR as a motivational tool during decision-making helps patients in making informed choices and, as a consequence, can promote the uptake of home dialysis over in-center therapies. The use of VR software could similarly improve the educational or training offer because the medical contents can be given in a standardized manner in every dialysis clinic.

**Figure 2:**
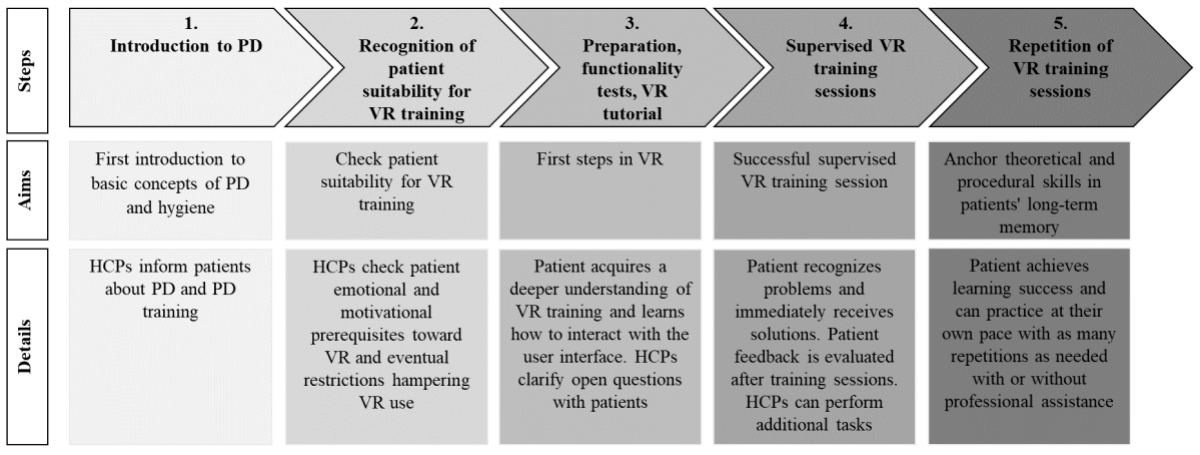
Process proposal to implement virtual reality (VR)–based peritoneal dialysis (PD) training programs. On the basis of the collected data, we propose a 5-step workflow that can serve as a guide for health care professionals (HCPs) and stakeholders to introduce the VR-based PD training within other dialysis centers: (1) HCPs’ assessment of patients’ suitability for home dialysis, (2) evaluation of patients’ attitude and suitability toward the VR technology, (3) pretraining VR tutorials, (4) supervised VR-based PD training sessions, and (5) repetition of the various activities based on individual educational needs.

On the basis of these observations, we developed a 5-step process proposal that can serve as a guide to implement VR-based PD training within dialysis centers (Figure 2): (1) introduction to PD; (2) recognition of the patient’s suitability for VR training; (3) preparation, functionality tests, and VR training tutorial; (4) supervised VR-based PD training sessions; and (5) repetition of VR-based PD training sessions. During the first step, HCPs evaluate patients’ psychological, emotional, and physical barriers toward home dialysis to determine whether the patient meets the requirements for PD treatment (step 1, Figure 2). Before starting VR-based training, patients’ attitude toward the technology as well as the presence of cognitive or physical disabilities potentially hampering the use of VR must be thoroughly evaluated (step 2, Figure 2). After checking equipment suitability, pretraining VR tutorials are administered, allowing patients to get familiar with the use of the VR tools (step 3, Figure 2). These preliminary sessions are intended to provide learners with all the instructions necessary to use the devices before starting PD training, with the ultimate goal to reduce patients’ cognitive load at later steps. Next, supervised VR-based PD training sessions can be started (step 4, Figure 2). HCPs can check patients’ understanding and eventually address any application-oriented questions directly. In addition, professionals evaluate patients’ training needs and assess whether obstacles to the learning process are present. This approach enables HCPs to not only customize the information delivery process but also improve patient empowerment and self-confidence. The final step involves the repetition of the learning activities based on individual educational needs (step 5, Figure 2). HCPs can eventually check the acquisition of the procedural skills and provide suggestions to reinforce patient engagement in training activities.

This study has some limitations. One of the limitations is the small sample size, which may limit the generalizability of our findings to a broader population. In addition, while qualitative interviews provide valuable insights and generate working hypotheses, such an approach may introduce potential biases and subjectivity in the data collection process. Furthermore, the study may not have captured the full range of perspectives and experiences. Future research with a larger sample size could provide a more comprehensive understanding about the use of VR training for dialysis.

### Conclusions

In conclusion, VR-based training is intended to facilitate and accelerate patients’ skill acquisition required to perform a real PD bag exchange by themselves. Using this innovative technology for PD training is well accepted and feasible and provides the potential for nephrology HCPs to reach a large population and promote and facilitate home dialysis uptake. Further research is required to investigate the long-term effects of VR training on patient satisfaction, infection rates, and the longevity of PD treatment.

## Appendix

Multimedia Appendix 1 Interview guide.

## Abbreviations

CAPD: continuous ambulatory peritoneal dialysis
CKD: chronic kidney disease
HCP: health care professional
PD: peritoneal dialysis
VR: virtual reality

## Data Availability

All data generated or analyzed during this study are included in this published article (Multimedia Appendix 1).
