# Supplementary material for: The Use of a Novel Virtual Reality Training Tool for Peritoneal Dialysis: Qualitative Assessment Among Health Care Professionals
Source: JMIR Med Educ. 2024 Aug 6;10:e46220. doi: 10.2196/46220 (PMC11336508; doi:10.2196/46220)
Supplement: Multimedia Appendix 1 [file mededu_v10i1e46220_app1.docx]

Multimedia Appendix 1. Interview guide

1. Introduction
2. Open section
   1. Prioritization and designation of topics
   2. Narrative part
   3. Discursive part
3. Structured section (questionnaire) with narrative and discursive parts
4. Conclusion

Information about the center should be requested in advance in writing using a small list of questions, e.g. staff and qualification structure or patient structure (age, language, previous experience PD, multimorbidity, limitations)

Epistemic question:

1. What are the problem situations in traditional training (in relation to specific patient groups)?
2. How can VR help solve them?
3. What added value can VR deliver?
   1. regarding the efficiency of the training?
   2. regarding the quality and effectiveness of the training?
   3. with a view to patient motivation and the fun factor in training?
4. How is VR implemented in the training process? What are the application or key situations here?

1. introduction: 10'

**Welcome and introduction**:

**Documentation and data protection**:

**Outline of the interview**: The interview contains 5 sections: ...

**Age**: Could you please tell us how old you are?

**Professional background**: What professional qualifications do you have? Do you have any additional qualifications? What is the focus of your experience?

**Length of service**: How long have you worked at this center?

**Areas of focus**: What are your current areas of focus?

**PD experience**: How long have you been working with PD patients?

**Number of PD patients cared for**: How many PD patients are you currently caring for?

**Percentage of work with PD:** What percentage (roughly speaking) do you work with PD patients?

2. open section: 20'

**Introductory question**: What have been **important experiences** for you so far with the new VR training?

**Derivation of topic areas**: What exactly is the theme here? Can they give a title to this experience?

**Prioritization question**: Let's rank these topics in order of importance or significance. Which topic has the highest importance to you? (etc.)

**Description and examples**: What exactly were your experiences with this topic? Do you have a concrete, perhaps also typical, experienced situation?

**Comprehension questions**: What exactly happened? What exactly was your experience? What else is part of this experience? What do you mean by that? May I repeat that in my own words?

**In-depth and background questions, hypothesis-driven questions**: How do you evaluate this? What is your opinion on this? What do you think are the causes of this? What are the consequences? What connections to ... do you see? What are your expectations? How would this be conceivable differently? What would be your ideal solution?

3. structured section (with standardized, open questions)

***Advantages and benefits of the use of VR in the center: (Focus 1)***

**Strengths of the classic training**: What went really well as it was? What was familiar? What was goal-oriented? What was well standardized and efficient?

**Problem areas in classical training**: What were the problem areas in classical training? What were the problem areas with specific patient groups?

**Goals associated with VR deployment**: What should go better with VR? How can VR help solve the problem areas mentioned? Where do you see a further benefit of using VR in training?

(**Potential) target groups for VR**: In which patient groups is VR training used so far? What could it look like in the future?

(**Potential) areas of use**: What are the areas of use for VR training right now? What might this look like in the future?

*Familiarization before / after:*

**Target groups, users**: Who conducted the classic VR training at your company? Has this been adopted for VR training or have there been changes?

**Skill requirements**: Are there any specific skill requirements on your end to deliver the traditional training? What are the requirements for VR training?

**Use to date**: How has VR been used internally for qualification to date?

**Idea for future use**: What should this look like in the future?

***Training process and structure before / after: (focus 2)***

**Time of use**: When is the VR training used with regard to the use of the catheter (before, after, other)? Has the timing of deployment changed compared to traditional training? How is this planned in the future?

**Description of the training process and repetitions as an example**: Please describe what a training process or a training week looks like for you? What happens beforehand? How does the training process start? How long does the briefing take? How long is the first training sequence? How does it continue after that? How often does the patient come to the center for this? How often is the training repeated? What, in particular, is repeated? When and with which process step does the training end?

**Time required for instruction and opportunity for other activities**: How involved are you through the training? Are there other activities that you can perform during the training? To what extent is this possible?

*Form and time requirements of the learning outcome assessment:*

**Degree of standardization**: In what form is the patient's learning success monitored? Are there standardized methods for this?

**Flexibility, customizability**: How much do you cater to the individual patient?

**Load HCPs**: What is the time commitment involved?

**Differences**: Does this change as a result of VR training?

*VR technology:*

**Manageability**: How do you see the manageability of the VR training? How easy is it for the patients?

**Difficulties** (in general, with specific groups): Where are difficulties? With which groups?

**Change requests**: What would you like to see differently? What is missing?

*VR content:*

**Didactic preparation**: How well are patients introduced to the topic and its handling? How comprehensible and easy to digest is the VR training?

**Methodological quality** (tutorial, error correction, subtitles, etc.): How helpful are the support elements, tutorial, correction of errors, subtitles, etc.? What do patients really use?

**Capture of the teaching content**: Is all content to be learned sufficiently mapped? What is missing?

**Complementarity**: From your point of view, what is the fit with the materials already available or used in the center? How well does the VR training fit into the existing processes?

**Motivational character**: To what extent does the play and fun factor help learning?

**Leadership character**: How well are patients guided through the learning process? How well does it work without your intervention? Where do you ever have to help?

*Effectiveness:*

**Patient satisfaction**: What is the feedback from patients? What is the range here?

**Learning success**: How do you rate the learning success in comparison to classic training? Where is it greater, where less?

**Risks**: What risks do you see in a full transition to VR?

*Efficiency:*

**Evaluation of the time required**: How does time commitment compare to traditional training?

**Ratio of effort to benefit**: Is the cost-benefit ratio appropriate?

**Patient throughput**: Can you care for more patients with the new method?

*Summary:*

**Strengths and weaknesses**: In your view, what are the strengths of VR training? Where are weaknesses and risks?

4. conclusion

Thanks, further procedure.
